# Supplementary material for: Heparin-based hydrogel scaffolding alters the transcriptomic profile and increases the chemoresistance of MDA-MB-231 triple-negative breast cancer cells
Source: Biomater Sci. 2020 Feb 13;8(10):2786–96. doi: 10.1039/c9bm01481k (PMC7497406; doi:10.1039/c9bm01481k)
Supplement: Supplementary file 2 [file BM-008-C9BM01481K-s002.zip › Supplementary File 4/HP-B hydrogel + EGF (s)vControl/Pathways/my_analysis.Gsea.1545201170987/HALLMARK_PANCREAS_BETA_CELLS.html]

Details for gene set HALLMARK\_PANCREAS\_BETA\_CELLS[GSEA]

|  || Dataset | expr.class.cls#HYDEGF\_versus\_CONTROL.class.cls#HYDEGF\_versus\_CONTROL\_repos |
| Phenotype | class.cls#HYDEGF\_versus\_CONTROL\_repos |
| Upregulated in class | CONTROL |
| GeneSet | HALLMARK\_PANCREAS\_BETA\_CELLS |
| Enrichment Score (ES) | -0.47162548 |
| Normalized Enrichment Score (NES) | -1.3752694 |
| Nominal p-value | 0.089613035 |
| FDR q-value | 0.045304183 |
| FWER p-Value | 0.491 |
Table: GSEA Results Summary

  

Fig 1: Enrichment plot: HALLMARK\_PANCREAS\_BETA\_CELLS      
 Profile of the Running ES Score & Positions of GeneSet Members on the Rank Ordered List

  

| PROBE | DESCRIPTION (from dataset) | GENE SYMBOL | GENE\_TITLE | RANK IN GENE LIST | RANK METRIC SCORE | RUNNING ES | CORE ENRICHMENT || 1 | CHGA | na |  |  | 661 | 1.939 | 0.0876 | No |
| 2 | PCSK1 | na |  |  | 3625 | 0.880 | -0.0114 | No |
| 3 | VDR | na |  |  | 4214 | 0.779 | 0.0070 | No |
| 4 | MAFB | na |  |  | 8063 | 0.234 | -0.1789 | No |
| 5 | FOXO1 | na |  |  | 8633 | 0.166 | -0.1981 | No |
| 6 | AKT3 | na |  |  | 10132 | 0.001 | -0.2761 | No |
| 7 | NKX6-1 | na |  |  | 10893 | -0.092 | -0.3099 | No |
| 8 | SYT13 | na |  |  | 11916 | -0.214 | -0.3497 | No |
| 9 | LMO2 | na |  |  | 12668 | -0.308 | -0.3695 | No |
| 10 | SRPRB | na |  |  | 14628 | -0.577 | -0.4353 | Yes |
| 11 | ELP4 | na |  |  | 14819 | -0.607 | -0.4070 | Yes |
| 12 | STXBP1 | na |  |  | 15061 | -0.644 | -0.3790 | Yes |
| 13 | DPP4 | na |  |  | 15076 | -0.647 | -0.3390 | Yes |
| 14 | SEC11A | na |  |  | 15723 | -0.769 | -0.3242 | Yes |
| 15 | FOXA2 | na |  |  | 17700 | -1.332 | -0.3434 | Yes |
| 16 | SPCS1 | na |  |  | 18510 | -1.845 | -0.2695 | Yes |
| 17 | SRP9 | na |  |  | 18819 | -2.228 | -0.1453 | Yes |
| 18 | SRP14 | na |  |  | 19015 | -2.621 | 0.0095 | Yes |
Table: GSEA details [plain text format]

  

Fig 2: HALLMARK\_PANCREAS\_BETA\_CELLS      
 Blue-Pink O' Gram in the Space of the Analyzed GeneSet

  

Fig 3: HALLMARK\_PANCREAS\_BETA\_CELLS: Random ES distribution      
 Gene set null distribution of ES for **HALLMARK\_PANCREAS\_BETA\_CELLS**
